# Hypoosmotic stress induces R loop formation in nucleoli and ATR/ATM-dependent silencing of nucleolar transcription

**DOI:** 10.1093/nar/gkz436

**Published:** 2019-05-22

**Authors:** Artem K Velichko, Nadezhda V Petrova, Artem V Luzhin, Olga S Strelkova, Natalia Ovsyannikova, Igor I Kireev, Natalia V Petrova, Sergey V Razin, Omar L Kantidze

**Affiliations:** 1 Laboratory of Genome Stability, Institute of Gene Biology, Russian Academy of Sciences, 119334 Moscow, Russia; 2 Institute for Translational Medicine and Biotechnology, Sechenov First Moscow State Medical University, 119991 Moscow, Russia; 3 A.N. Belozersky Institute of Physico-Chemical Biology, Moscow State University, 119992 Moscow, Russia; 4 V.I. Kulakov National Medical Research Center for Obstetrics, Gynecology, and Perinatology, 117997 Moscow, Russia; 5 Laboratory of Structural and Functional Organization of Chromosomes, Institute of Gene Biology, Russian Academy of Sciences, 119334 Moscow, Russia; 6 Department of Molecular Biology, Moscow State University, 119234 Moscow, Russia; 7 LFR2O, Institute Gustave Roussy, F-94805 Villejuif, France

## Abstract

The contribution of nucleoli to the cellular stress response has been discussed for over a decade. Stress-induced inhibition of RNA polymerase I-dependent transcription is hypothesized as a possible effector program in such a response. In this study, we report a new mechanism by which ribosomal DNA transcription can be inhibited in response to cellular stress. Specifically, we demonstrate that mild hypoosmotic stress induces stabilization of R loops in ribosomal genes and thus provokes the nucleoli-specific DNA damage response, which is governed by the ATM- and Rad3-related (ATR) kinase. Activation of ATR in nucleoli strongly depends on Treacle, which is needed for efficient recruitment/retention of TopBP1 in nucleoli. Subsequent ATR-mediated activation of ATM results in repression of nucleolar transcription.

## INTRODUCTION

The ability of cells to sense DNA lesions and initiate the DNA damage response (DDR) is a prerequisite for maintaining genome stability. In higher eukaryotes, the ataxia telangiectasia mutated (ATM) and the ATM- and Rad3-related (ATR) kinases are two crucial players orchestrating the DDR. Along with the related DNA-dependent protein kinase catalytic subunit (DNA-PKcs), encoded by the *PRKDC* gene, ATM and ATR belong to the PI3K-like kinase (PIKK) family (1). DNA-PKcs and ATM are primarily activated by DNA double-stranded breaks (DSBs) with the assistance of DSB sensor factors, such as the Ku70/80 heterodimer and the Mre11-Rad50-Nbs1 (MRN) complex (2). ATR initiates the DDR in response to single-stranded DNA (ssDNA) produced by alterations in a broad spectrum of cellular processes (3). ATR activation also requires auxiliary factors, with ATRIP and TopBP1 being the most important of these (3). Although ATM and ATR share many of their phosphorylation targets, the DDR signaling pathways that they guide are generally self-sufficient and rarely interconnected. However, ATM can act upstream of ATR, particularly in cases when the progressive resection of DSBs is promoted (4−5). Experimental data describing the reverse situation are not as abundant; however, it was reported that ATM could be activated by ATR-mediated phosphorylation (6).

Nucleoli, the largest subnuclear compartments, are formed around arrays of ribosomal gene repeats (rDNA) and transcribed by RNA polymerase I (Pol I) to produce pre-ribosomal RNA (7). The primary function of nucleoli, ribosome biogenesis, consumes considerable cellular energy. It was previously established that the nucleolus could coordinate the cell stress response in a number of ways (7). Thus, many nucleolar proteins are known to move from nucleoli and regulate cellular processes, such as DNA replication recovery and p53 activation, under stress conditions (8). Alternatively, genotoxic stress inhibits Pol I-dependent transcription of rDNA (9,10). At present, most experimental evidence suggests that such transcriptional silencing accompanies only DNA damage, induced in rDNA (11,12). However, a limited number of studies reported the possibility of *in trans* inhibition of Pol I-dependent transcription by DNA lesions, arising outside nucleoli (13). It is generally proposed that the DNA damage-induced silencing of rDNA transcription relies on the activity of ATM (11−13). Nucleolar DNA damage is accompanied by ATM-dependent relocalization of rDNA to the so-called ‘caps’ at the periphery of nucleoli (10). It is suggested that such rDNA clustering facilitates the homologous recombination (HR) repair of DSBs, induced in rDNA sequences; however, it is questionable whether the formation of nucleolar caps is necessary for transcriptional silencing. The mechanism by which ATM inhibits Pol I-dependent transcription and whether it is a key player in this process remain poorly understood.

Here, we demonstrate that under hypoosmotic stress conditions R loops in transcribed ribosomal genes are stabilized, thus, generating RPA-coated stretches of ssDNA. This leads to the recruitment of ATR to nucleoli and its ATRIP- and TopBP1-dependent activation. Interestingly, Treacle (TCOF1) is indispensable for TopBP1 recruitment/retention in nucleoli and proper activation of ATR. Subsequent activation of ATM is mediated by ATR and does not depend on DSBs. Finally, ATM shuts down nucleolar transcription possibly through one of the known mechanisms of ATM-dependent Pol II transcription silencing (14−15).

## MATERIALS AND METHODS

### Antibodies

The primary antibodies used for immunofluorescence and/or western blot hybridization were H2AX (rabbit, Cell Signaling, #7631), γH2AX (mouse, Millipore, #05-636), cyclin B1 (rabbit, Santa Cruz Biotechnology, #sc-752), histone H3 (rabbit, Abcam, #ab1791), cyclin E1 (mouse, Cell Signaling, #4129), ATM (rabbit, Genetex, #GTX111106), pATM (rabbit, Abcam, #ab81292), pATM (mouse, Cell Signaling, #4526), ATR (rabbit, Genetex, #GTX128146), pATR (rabbit, Cell Signaling, #58014), Nbs1 (rabbit, Abcam, #ab23996), pNbs1 (rabbit, Cell Signaling, #3001), pChk1 (rabbit, Cell Signaling, #2341), pChk2 (rabbit, Cell Signaling, #2197), pRPA (rabbit, Bethyl, #A300-246A), TopBP1 (mouse; Santa Cruz Biotechnology, #sc-271043), Mre11 (rabbit, Novus Biologicals, #Nb100-142), B23 (mouse, Sigma, #B0556), Nucleolin (rabbit, Sigma, #N2662), UBF (mouse, Santa Cruz Biotechnology, #sc-13125), Ki67 (rabbit, Cell Signaling, #9129), CHD4 (mouse, Abcam, #ab70469), Treacle/TCOF1 (rabbit, Sigma, #HPA038237), S9.6 (mouse, Millipore, #MABE1095), BrdU (rabbit, Rockland Immunochemicals, #600-401-C29). The secondary antibodies conjugated to either Alexa Fluor 488 or Alexa Fluor 594 were purchased from Molecular Probes/Life Technologies; the horseradish peroxidase-conjugated anti-mouse and anti-rabbit IgG were purchased from Amersham/GE Healthcare. The specificity of phospho-specific antibodies was studied using HeLa cells depleted for corresponding factors (Supplementary Figure S1).

### Cell culture and synchronization

Human HeLa (ATCC^®^ CCL-2™), HT1080 (ATCC®CCL-121™), HEK293 (ATCC^®^ CRL-1573™) and mouse CT26 (ATCC^®^ CRL-2638™) cells were cultured in Dulbecco's modified Eagle's medium (DMEM) (PanEco) supplemented with 10% fetal bovine serum (FBS; HyClone/GE Healthcare) and penicillin/streptomycin. Human skin fibroblasts (female 46XX) were cultured in DMEM (PanEco) supplemented with 10% FBS (HyClone/GE Healthcare) and with 10 ng/ml fibroblasts growth factor. The cells were cultured at 37°C in a conventional humidified CO_2_ incubator.

For G1-phase synchronization, the cells were treated with 2 mM thymidine for 16 h, released for 8 h from the block and then treated with 30 ng/ml nocodazole (Sigma) for 12 h. Mitotic cells were harvested by mechanical shake-off, washed extensively in phosphate-buffered saline (PBS), and replated at 37°C for 3–5 h.

For S-phase synchronization, the cells were treated with 2 mM thymidine for 16 h. To release the cells from thymidine, they were washed twice with PBS and the drug-free medium was added for 2–3 h.

For G2-phase synchronization, the cells were treated with 2 mM thymidine for 16 h, released for 8 h from the block and then treated with 2 mM thymidine for an additional 16 h. To release the cells from double thymidine, they were washed twice with PBS and the drug-free medium was added for 8 h.

### Drug treatment and osmotic stress

Hypoosmotic stress was applied by incubation the cells in 50% DMEM/50% H2O for 30 min–3 h. Hyperosmotic stress was applied by incubation in DMEM supplemented with 600 mM NaCl for 1 h. For kinase inhibition experiments, cells were treated with 20 μM KU55933 (Tocris Bioscience) for 3 h, 15 μM VE821 (Sigma) for 3 h or 50 μM NU7026 (Adooq Bioscience) for 6 h. For transcription inhibition experiments, cells were treated with 0.01 μg/ml actinomycin D (Biotium) for 3 h, or 50 μM DRB (Santa Cruz Biotechnology) for 3 h. For induction of DSBs, the cells were treated with 1–20 μg/ml etoposide (Sigma) for 1 h. I-PpoI nuclear translocation was initiated by incubation the cells with 5 μM 4-hydroxytamoxifen (4-OHT) (Sigma) for 4–16 h.

### Gene knockdown

RNA interference experiments were performed using Dharmafect siRNA transfection reagent (Thermo Scientific) following the manufacturer's instructions. The cells were transfected with 200 nM ATM siRNA (Santa Cruz Biotechnology, #sc-29761), 50 nM ATR siRNA (Santa Cruz Biotechnology, # sc-29763), 100 nM DNA-PKcs (PRKDC; Santa Cruz Biotechnology, #sc-35200), 50 nM Nbs1 siRNA (Santa Cruz Biotechnology, # sc-36061), 50 nM ATMIN siRNA (Santa Cruz Biotechnology, # sc-105098), 50 nM CHD4 siRNA (Santa Cruz Biotechnology, #sc-37953), 50 nM Treacle/TCOF1 (Santa Cruz Biotechnology, #sc-61707). For MTA2 and ATRIP knockdowns, the cells were transfected with 50 nM siRNAs designed by BLOCK-iT RNAi Designer (Thermo Scientific). Sequences of custom-made siRNAs used in the study are provided in a Supplementary Table S1. Forty-eight hours after transfection, the cells were harvested for further analysis.

For CRISPR/Cas9-mediated knockout, two single guide RNAs (sgRNA) flanking a region of the target gene (*H2AX* or *TOPBP1*) were designed using the guide RNA design tool (www.atum.bio/eCommerce/cas9/input). The sgRNA targeting sequences were separately cloned into the sgRNA/Cas9 expression vector pSpCas9n(BB)-2A-Puro (PX462) V2.0 (Addgene #62987). A list of all oligonucleotides is provided in Supplementary Table S2. The plasmids were co-transfected into HeLa cells with X-fect transfection reagent (Clontech Laboratories). The transfectants were selected with 10 μg/ml puromycin for 24 h. After 24 h of puromycin selection, cells were switched to their normal culture medium. Clones of HeLa cells were obtained by limiting dilution into 96-well plates. Western blotting and indirect immunofluorescence was used to identify clones with H2AX or TopBP1 depletion.

Knockdown/knockout efficiency was analyzed by Western blotting or qPCR-based gene expression analysis (Supplementary Figure S2).

### Neutral comet assay

After treatment, cells were trypsinized with 0.25% trypsin for several minutes at 37°C. The trypsin was inactivated with a 4-fold volume of DMEM. Cell suspension at a concentration of 10^5^ cells/ml was mixed in a 1:1 ratio with Trevigen LMAgarose (#4250-050-02) at 37°C. The mixture was pipetted onto comet slides (Trevigen, #3950-300-02) that had been pre-coated with a 1% normal melting point agarose (Sigma) base layer. The drop containing the cells was covered with a glass cover slip and incubated at 4°C for 5 min. After incubation, the cover slips were removed, and the slides were immersed in lysis solution (30 mM ethylenediaminetetraacetic acid (EDTA), 0.5% sodium dodecyl sulphate (SDS) and 10 mM Tris–HCl, pH 8.0, supplemented with 500 μg/ml proteinase K) and incubated at 37°C for 1 h. After lysis, the slides were washed three times for 5 min in PBS and incubated in 1× TBE (Tris-Borate-EDTA buffer) for 20 min at 4°C. Electrophoresis was performed in a Trevigen electrophoresis system (#4250-050-ES) for 10 min at 4°C and 1 V/cm in 1× TBE. The comets were counterstained with SYBR Green for 1 h (1:3000; Thermo Scientific, #S7563). The comets were visualized at four magnification using an inverted Nikon Eclipse Ti-E fluorescence microscope equipped with a Nikon Intensilight C-HGFI light source (objective: Nikon Plan Fluor 4/0.13; camera: DS-Qi2). The images of the comets were analyzed using CellProfiler software (version 2.1.1 rev 6c2d896).

### Whole-cell extracts preparation and immunoblotting

Cells were lysed by incubation in RIPA buffer (150 mM NaCl, 1% Triton X-100, 0.5% sodium deoxycholate, 0.1% SDS, 50 mM Tris–HCl (pH 8.0) supplemented with Protease Inhibitor Cocktail (Bimake) and Phosphatase Inhibitor Cocktail (Bimake) for 30 min on ice. Next, the cell extracts were sonicated with a VirSonic 100 ultrasonic cell disrupter and stored at −70°C. The protein concentration was measured by the Bradford assay. Aliquots of each sample were separated by sodium dodecyl sulphate-polyacrylamide gelelectrophoresis and blotted onto polyvinylidene difluoride (PVDF) membranes (Amersham/GE Healthcare). The membranes were blocked for 1 h in 2% ECL Advance blocking reagent (GE Healthcare) or 2% bovine serum albumin (BSA) (Sigma) in PBS containing 0.1% Tween 20 (PBS-T) followed by incubation overnight at 4°C with a primary antibody diluted in PBS-T containing 2% blocking reagent or 2% BSA. After three washes with PBS-T, the membranes were incubated for 1 h with the secondary antibodies (horseradish peroxidase-conjugated anti-rabbit or anti-mouse IgG) in PBS-T containing 2% blocking agent or 2% BSA. The immunoblots were visualized using a Pierce ECL plus western blotting substrate.

### Immunofluorescence microscopy

For immunostaining, cells were grown on microscope slides. All samples were fixed in CSK buffer (10 mM PIPES, pH 7.0, 100 mM NaCl, 1.5 mM MgCl_2_, 300 mM sucrose) supplemented with 1% paraformaldehyde (PFA) and 2.5% Triton X-100 for 15 min at room temperature or in 100% cold methanol (−20°C) for 10 min. After washing in PBS, the cells were pre-incubated with 1% BSA in PBS with 0.05% Tween 20 for 30 min and were then incubated with antibodies in PBS supplemented with 1% BSA and 0.05% Tween 20 for 1 h at room temperature or overnight at 4°C. After incubation, the cells were washed three times (5 min each time) with PBS supplemented with 0.2% BSA and 0.05% Tween 20. The primary antibodies bound to antigens were visualized using Alexa Fluor 488- or Alexa Fluor 594-conjugated secondary antibodies. The DNA was counterstained with the fluorescent dye 4,6-diamino-2-phenylindole (DAPI) for 10 min at room temperature. The samples were mounted using Dako fluorescent mounting medium (Life Technologies). The immunostained samples were analyzed using a Zeiss AxioScope A.1 fluorescence microscope (objectives: Zeiss N-Achroplan 40 × /0.65 and EC Plan-Neofluar 100 × /1.3 oil; camera: Zeiss AxioCam MRm; acquisition software: Zeiss AxioVision Rel. 4.8.2; Jena, Germany). The images were processed using ImageJ software (version 1.44). The images were analyzed using CellProfiler software (version 2.1.1 rev 6c2d896).

Samples for Structured Illumination Microscopy (SIM) were mounted in Dako fluorescent mounting medium (Life Technologies) and examined using a Nikon N-SIM microscope (100×/1.49 NA oil immersion objective, 488 nm and 561 nm diode laser excitation). Image stacks (z-steps of 0.2 μm) were acquired with EMCCD camera (iXon 897, Andor, effective pixel size 60 nm). Exposure conditions were adjusted to get a typical yield about 5000 max counts (16-bit raw image) while keeping bleaching minimal. Image acquisition, SIM image reconstruction and data alignment were performed using NIS-Elements (Nikon).

### Immunostaining of RNA:DNA hybrids with S9.6 antibody

For S9.6 staining, the cells were fixed in 100% cold methanol (−20°C) for 10 min, washed three times with PBS, and treated with RNase A (50 μg/ml) in a buffer containing 5 mM EDTA, 400 mM NaCl, 10 mM Tris–HCl (pH 7.5) for 1 h at 37°C (16). For negative control after RNase A treatment cells were treated additionally with RNase H (Roche) for 3 h at 37°C. After washing in PBS, the cells were preincubated with 1% BSA in PBS with 0.05% Tween 20 for 30 min and were incubated with S9.6 antibody (mouse, Millipore, #MABE1095) in PBS supplemented with 1% BSA and 0.05% Tween 20 for overnight at 4°C and the standard immunofluorescence protocol was followed.

### Gene expression analysis

RNA was extracted from cells using TRIzol reagent (Life Technologies). All RNA samples were further treated with DNase I (Thermo Scientific) to remove the residual DNA. RNA (1 μg) was reverse transcribed in a total volume of 20 μl for 1 h at 42°C using 0.4 μg of random hexamer primers and 200 U of reverse transcriptase (Thermo Scientific) in the presence of 20 U of ribonuclease inhibitor (Thermo Scientific). *PAPAS* cDNA was synthesized with rDNA-specific primers fused to the T7 promoter.

The cDNA obtained was analyzed by quantitative polymerase chain reaction (qPCR) using the CFX96 real-time PCR detection system (Bio-Rad Laboratories). The PCRs were performed in 20 μl reaction volumes that included 50 mM Tris–HCl (pH 8.6), 50 mM KCl, 1.5 mM MgCl_2_, 0.1% Tween-20, 0.5 μM of each primer, 0.2 mM of each dNTP, 0.6 μM EvaGreen (Biotium), 0.75 U of Hot Start Taq Polymerase (Sibenzyme) and 50 ng of cDNA. *PAPAS* cDNA was amplified by PCR using a T7 forward primer and an rDNA-specific reverse primer. Primers used in the study are listed in Supplementary Tables S3 and 4.

### Cell viability

Cells were seeded at 1 × 10^4^ in a 96-well plate. MTT (3-(4,5-dimethylthiazol-2-yl)-2,5-diphenyltetrazolium bromide) was added to 200 μM final concentration and incubated at 37°C for 3 h. After that cells were lysed in 100% DMSO (dimethyl sulfoxide). The plate was read at 590 nm using a Synergy H4 Hybrid Multi-Mode Microplate Reader.

Caspases activity was measured using CellEvent Caspase-3/7 Detection Reagent (Thermo Scientific) according to the manufacturer's recommendations.

### Replication and transcription

For 5-ethynyl-2′-deoxyuridine (EdU) incorporation, the cells were incubated with 10 μM EdU (Life Technologies) for 0.5–1 h at 37°C. Then, the cells were washed three times with PBS and fixed in CSK buffer (10 mM PIPES, pH 7.0, 100 mM NaCl, 1.5 mM MgCl_2_, 300 mM sucrose) supplemented with 1% paraformaldehyde (PFA) and 2.5% Triton X-100 for 15 min at room temperature. The samples were then processed using a Click-iT EdU Imaging Kit (Life Technologies) according to the manufacturer's recommendations.

For 5-fluorouridine (FU) incorporation, the cells were incubated with 5 mM FU (Sigma) for 1–3 h at 37°C. After this incubation, the cells were washed three times with PBS and fixed in 100% cold methanol (−20°C) for 10 min before staining. The cells were washed three times with PBS and incubated with rabbit polyclonal anti-BrdU antibodies (Rockland Immunochemicals, #600-401-C29) in PBS supplemented with 1% BSA and 0.05% Tween 20 for 1 h at room temperature. The primary antibody was revealed using Alexa Fluor 488-conjugated goat anti-rabbit antibody as described above.

### Chromatin immunoprecipitation (ChIP)

Cells were fixed for 15 min with 1% formaldehyde at room temperature and crosslinking was quenched by adding 125 mM glycine for 5 min. Cells were harvested in PBS and nuclei were prepared by incubation in buffer FL (5 mM PIPES, pH 8.0, 85 mM KCl, 0.5% NP40 supplemented with Protease Inhibitor Cocktail (Bimake) and Phosphatase Inhibitor Cocktail (Bimake) for 30 min on ice. Next, chromatin was sonicated in RIPA buffer (10 mM Tris–HCl, pH 8.0, 140 mM NaCl, 1% Triton X-100, 0.1% Na-deoxycholate, 0.1% SDS) with a VirSonic 100 to an average length of 200–500 bp. Per chromatin immunoprecipitation (ChIP) reaction, ∼10–20 μg chromatin was incubated with 2–4 μg antibodies overnight at 4°C. Immunoprecipitations were performed using antibodies against ATM (mouse, Sigma, #A1106), γH2AX (mouse, Millipore, #05-636), Mre11 (rabbit, Novus Biologicals, #Nb100-142), TopBP1 (mouse, Santa Cruz Biotechnology, #sc-271043), Nbs1 (rabbit, Abcam, #ab23996), RPA (mouse, Abcam, #ab2175). Rabbit IgG (Jackson ImmunoResearch, #011-000-002) was used as a negative ChIP control. On the next day, Protein A/G Magnetic Beads (Thermo Scientific) were added in each sample and incubated for 4 h at 4°C. Immobilized complexes were washed two times for 10 min at 4°C in low salt buffer (20 mM Tris–HCl, pH 8.0, 150 mM NaCl, 2 mM EDTA, 0.1% SDS, 1% Triton X-100) and high salt buffer (20 mM Tris–HCl, pH 8.0, 500 mM NaCl, 2 mM EDTA, 0.1% SDS, 1% Triton X-100). Samples were incubated with RNase A (Thermo Scientific) for 30 min at room temperature. The DNA was eluted from the beads and decrosslinked by proteinase K digestion for 4 h at 55°C and subsequent incubation at 65°C for 12 h. Next, the DNA was purified using phenol/chloroform extraction and analyzed by qPCR. The qPCR primers used for ChIP analysis are listed in a Supplementary Table S5.

### S9.6 chromatin immunoprecipitation

Cells were fixed for 15 min with 1% formaldehyde at room temperature and crosslinking was quenched with 125 mM glycine for 5 min. Cells were harvested in PBS and nuclei were isolated by incubation in FL buffer (5 mM PIPES, pH 8.0, 85 mM KCl, 0.5% NP40 supplemented with Protease Inhibitor Cocktail (Bimake) and Phosphatase Inhibitor Cocktail (Bimake) for 30 min on ice. Сhromatin was sonicated in RIPA buffer (10 mM Tris–HCl, pH 8.0, 140 mM NaCl, 1% Triton X-100, 0.1% Na-deoxycholate, 0.1% SDS) with a VirSonic 100 ultrasonic cell disrupter to an average length of 300–500 bp. Next, RNase A and NaCl were added to final concentration 50 μg/ml and 400 mM, respectively, and incubated for 3 h at 37°C. For negative control 20U RNase H (Roche) was added to chromatin and incubated for 3 h at 37°C and then NaCl was added to 400 mM final concentration. To stop RNase reaction 500U RiboLock RNase inhibitors (Thermo) was added to all samples. Per immunoprecipitation reaction, ∼10–20 μg of prepared chromatin were incubated with 5 μg S9.6 antibody overnight at 4°C. On the next day, Protein A/G Magnetic Beads (Thermo Scientific) were added in each sample and incubated for 4 h at 4°C. Immobilized complexes were washed two times for 10 min at 4°C in low salt buffer (20 mM Tris–HCl, pH 8.0, 150 mM NaCl, 2 mM EDTA, 0.1% SDS, 1% Triton X-100) and high salt buffer (20 mM Tris–HCl, pH 8.0, 500 mM NaCl, 2 mM EDTA, 0.1% SDS, 1% Triton X-100). The DNA was eluted from the beads and decrosslinked by Proteinase K digestion for 4 h at 55°C and overnight at 65°C. Next, the DNA was purified using phenol/chloroform extraction and analyzed by qPCR. The qPCR primers used to analyze ChIP DNA are included in Supplementary Table S5.

### Statistical analysis

ChIP and RT-PCR data are reported as mean values from at least three biological replicates, with error bars denoting SD. Immunostaining and comet assay images were analyzed by CellProfiler software; the measurements obtained are presented in box-whisker plots. At least 500 cells were analyzed in each experiment. Comparisons between two groups were performed using a paired two-tailed Student's *t*-test using IBM SPSS Statistics 20.

## RESULTS

### Hypoosmotic stress induces ATR- and ATM-dependent phosphorylation of H2AX

**Figure 1.**
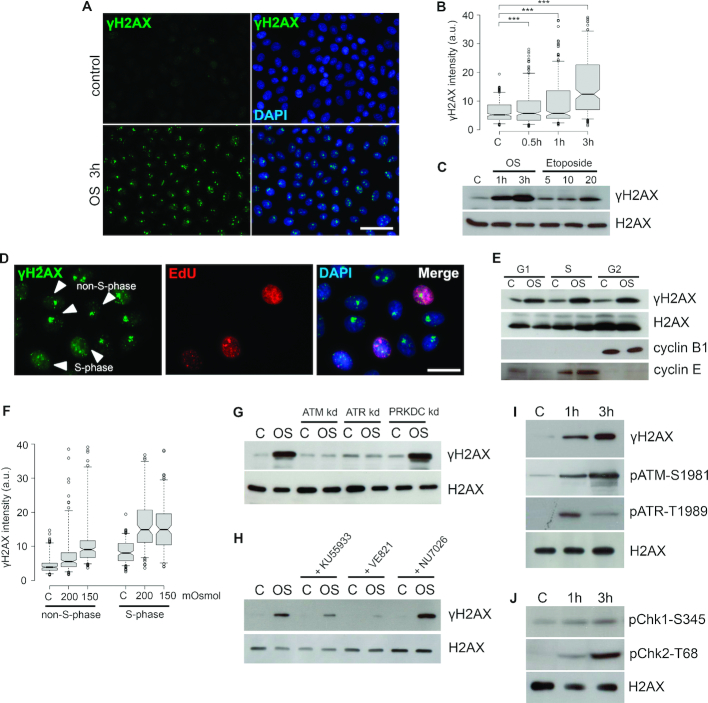
Hypoosmotic stress induces ATR and ATM-dependent phosphorylation of H2AX. (**A**) Human HeLa cells, untreated (control) or treated with hypoosmotic stress (OS) for 3 h were stained for γH2AX (green). The DNA was stained with DAPI (blue). Scale bar: 80 μm. (**B**) Quantification of γH2AX in control HeLa cells and cells subjected to hypoosmotic stress for the indicated time periods (0.5, 1 or 3 h). Box plots show the γH2AX fluorescence intensities. Horizontal lines represent the median. ***P < 0.0001, n.s.—not significant (unpaired *t*-test, *n* > 500). (**C**) HeLa cells were subjected to hypoosmotic stress for the indicated time periods (1 or 3 h) or treated with the indicated concentrations (5, 10 or 20 μg/ml) of the topoisomerase II poison etoposide for 1 h. WB was performed with an anti-γH2AX antibody. Throughout the figure, unmodified histone H2AX is used as a loading control in WB, and the control (C) represents untreated HeLa cells. (**D**) HeLa cells were pulse-labeled with EdU (10 μM, 30 min), subjected to hypoosmotic stress for 3 h and stained for γH2AX (green). EdU was revealed by Click Chemistry (red). The DNA was stained with DAPI (blue). Scale bar: 20 μm. (**E**) G1-, S- and G2-phase HeLa cells were subjected to hypoosmotic stress (OS) for 1 h. WB was performed with antibodies against γH2AX, cyclin B1 (a marker of G2 cells), and cyclin E (a marker of S cells). (**F**) HeLa cells were pulse-labeled with EdU (10 μM, 30 min), subjected to hypoosmotic stress (200 and 150 mOsm/l, 3 h), and stained for γH2AX. EdU was revealed by Click Chemistry. The DNA was stained with DAPI. Microscopic images were processed with CellProfiler software as follows: nuclei were segmented based on DAPI fluorescence, the cell population was divided into S-phase (EdU-positive) and non-S-phase (EdU-negative) cells and the γH2AX fluorescence intensity was measured. Box plots show the γH2AX fluorescence intensities. Horizontal lines represent the median. ***P < 0.0001, n.s.—not significant (unpaired *t*-test, *n* > 500). (**G** and **H**) WB analysis of γH2AX in HeLa cells pre-treated with siRNAs (**G**) or chemical compounds (**H**) to suppress the activity of either ATM (ATM kd and KU55933), ATR (ATR kd and VE821) or DNA-PKcs (PRKDC kd and NU7026) and subjected to hypoosmotic stress for 1 h. (**I** and **J**) HeLa cells were subjected to hypoosmotic stress for 1 or 3 h. WB was performed with antibodies against γH2AX, phospho-ATM (Ser1981), and phospho-ATR (Thr1989), phospho-CHK1 (Ser345), and phospho-CHK2 (Thr68).

In contrast to hyperosmotic stress, which is known to induce DSBs and phosphorylation of histone H2AX (γH2AX) (17), DNA damage under hypoosmotic stress conditions was not reported. To investigate this possibility, we used mild hypoosmotic stress, applied to cultured cells. We found that hypoosmotic stress induced prominent phosphorylation of histone H2AX in human HeLa cells, although this phosphorylation was not uniform across the cell population (Figure 1A). This phenomenon also occurred for several other human transformed and normal cell lines, namely fibrosarcoma cell line HT1080, transformed embryonic kidney cells HEK293 and skin normal fibroblasts, as well as the mouse colon carcinoma cell line CT26 (Supplementary Figure S3A). The level of H2AX phosphorylation directly depended on the duration of hypoosmotic stress and was significantly higher than one induced by a DSB-inducing agent, the topoisomerase II poison, etoposide (Figure 1B and C). Notably, the stress conditions used did not induce apoptosis or affect cell viability (Supplementary Figure S3B and C). The non-uniform distribution of γH2AX in an asynchronous cell population led us to hypothesize that distinct γH2AX patterns are somehow associated with the cell-cycle stage. Indeed, nearly pan-nuclear staining of γH2AX was typical for S-phase cells, whereas G1 and G2 cells contained a countable number of large γH2AX foci (Figure 1D). We found that γH2AX partially co-localized with replication foci in S cells (Supplementary Figure S3D), likely indicating that the hypoosmotic conditions used could result in DNA replication stress. Using G1, S and G2 cells, we verified that H2AX phosphorylation was efficiently induced by hypoosmotic stress throughout the cell cycle (Figure 1E), although more prominent γH2AX levels reached at milder hypoosmotic stress conditions were typical for S-phase cells (Figure 1F). Since the mechanisms of osmotic stress-induced replication arrest were out of the scope of the present study, we focused on replication-independent γH2AX phosphorylation and used G1-synchronized cells in most of the following experiments.

Next, we identified the PIKK responsible for H2AX phosphorylation upon hypoosmotic treatment. Using specific PIKK inhibitors and RNA interference, we found that γH2AX production depended on the activity of both ATR and ATM kinases (Figure 1G and H; Supplementary Figure S3E). Correspondingly, ATR and ATM were activated under hypoosmotic conditions, as indicated by the appearance of their phosphorylated forms (Figure 1I) and phosphoforms of target checkpoint kinases (Figure 1J) in response to mild hypoosmotic stress. Notably, the time course of the PIKKs activation differed significantly. The phospho-ATR levels reached a maximum during the first hour of treatment and later decreased, whereas ATM phosphorylation gradually increased during the course of the 3 h-long hypoosmotic stress (Figure 1I).

### Hypoosmotic stress-induced DNA damage response is nucleoli specific

**Figure 2.**
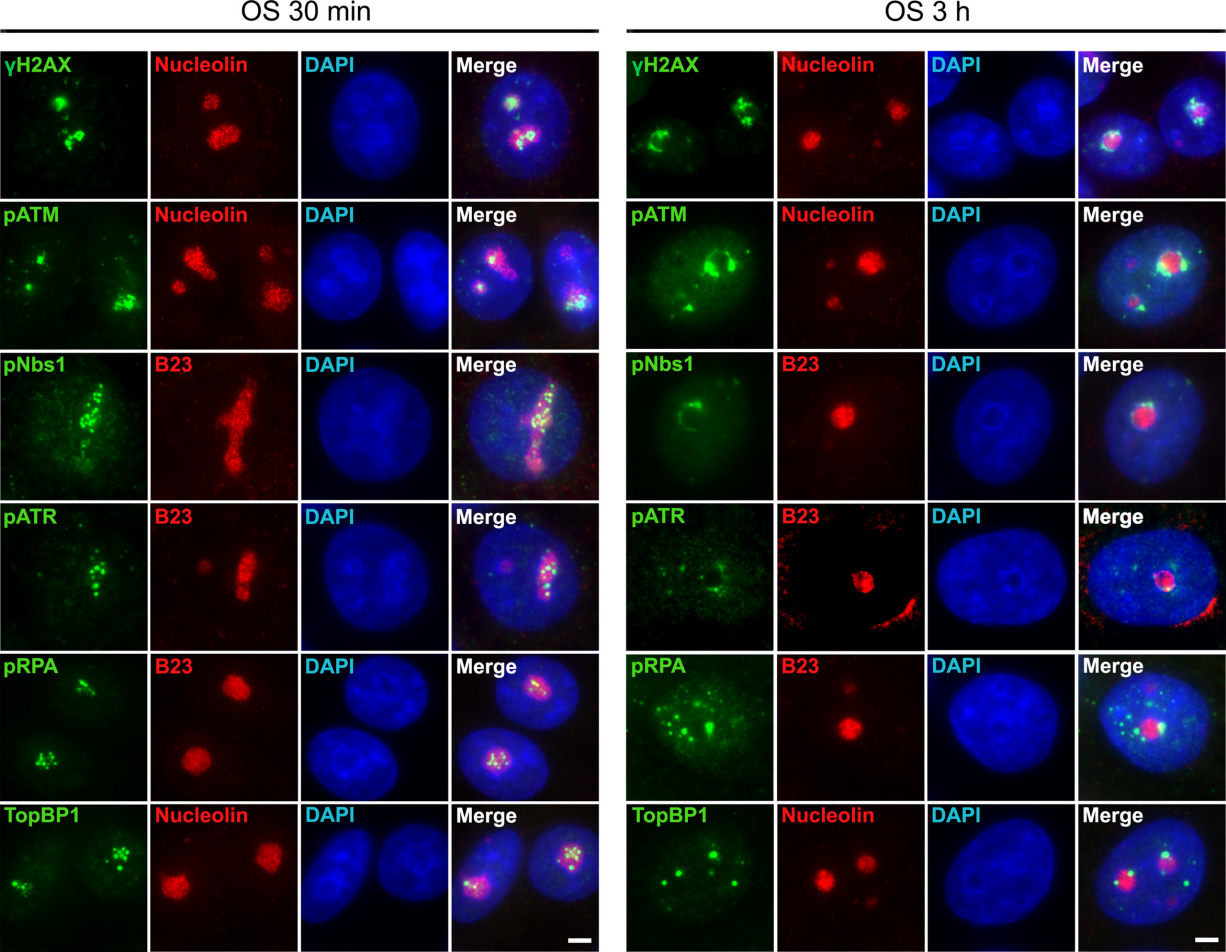
Hypoosmotic stress-induced DNA damage is nucleoli-specific. HeLa cells were subjected to hypoosmotic stress (OS) for 30 min or 3 h and stained for γH2AX, phospho-ATM (pATM; Ser1981), phospho-ATR (pATR; Thr1989), phospho-Nbs1 (pNbs1; Ser343), TopBP1 or phospho-RPA (pRPA; Ser33). In each case nucleoli were visualized by immunostaining the cells with antibodies against nucleolar marker proteins (nucleolin or B23). The DNA was stained with DAPI (blue). Scale bar: 5 μm.

**Figure 3.**
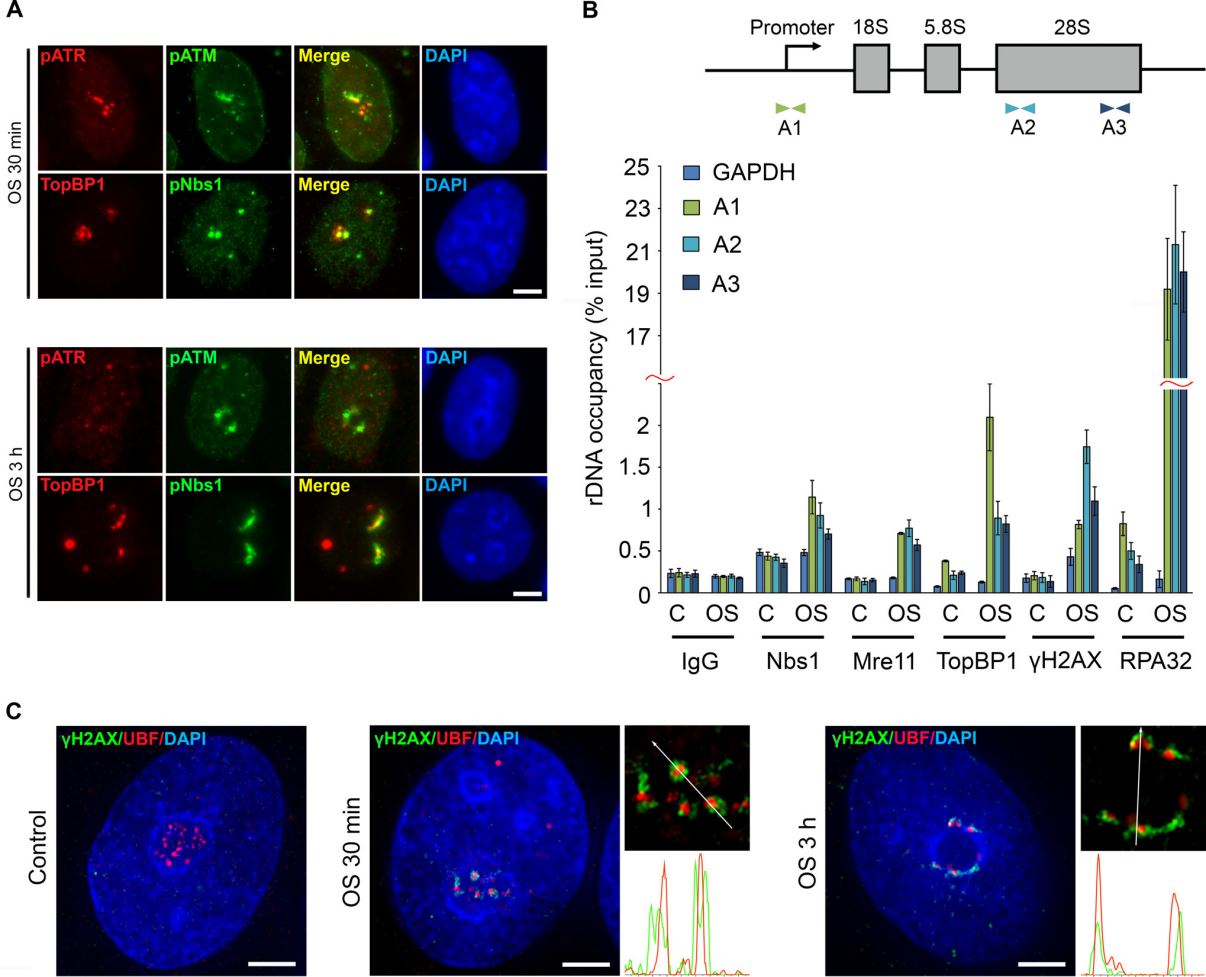
Hypoosmotic stress-induced DDR originates from actively transcribed ribosomal genes. (**A**) HeLa cells were subjected to hypoosmotic stress (OS) for 1 or 3 h and co-stained either for phospho-ATM (pATM; Ser1981) and phosphoATR (pATR; Thr1989) or for TopBP1 and phospho-Nbs1 (pNbs1; Ser343). The DNA was stained with DAPI (blue). Scale bar: 5 μm. (**B**) Occupancy of DNA repair factors at rDNA upon hypoosmotic stress (1 h). ChIP with IgG and antibodies against Nbs1, Mre11, TopBP1, RPA32 and γH2AX followed by qPCR using the rDNA amplicons positioned as indicated on the scheme above (A1: promoter, A2: 5′-28S rRNA region, A3: 3′-28S rRNA region; the amplicon from the GAPDH gene was used as a negative control). Data are represented relative to the input. Values are the means ± SD from at least three independent replicates. (**C**) HeLa cells were subjected to hypoosmotic stress (OS) for 30 min or 3 h and stained with antibodies against γH2AX (green) and UBF (red). Control represents HeLa cells that were not exposed to hypoosmotic stress. The DNA was stained with DAPI (blue). Structured illumination microscopy (SIM) analysis was performed. The co-localization analysis was performed on the merged images. The graphs illustrate the quantification in arbitrary units of the distribution of UBF and γH2AX fluorescence along the lines shown in the merged panels. Scale bar: 5 μm.

We analyzed the spatial and temporal distribution of DNA repair factors during hypoosmotic stress treatment. In G1 cells, hypoosmotic stress-induced phosphorylation of H2AX was associated with nucleoli (Figure 2 and Supplementary Figure S4A). The same localization was observed for major factors involved in ATR- (phospho-RPA, TopBP1, phospho-ATR) and ATM-dependent (phospho-Nbs1, phospho-ATM) DDR pathways (Figure 2 and Supplementary Figure S4B). In response to hypoosmotic treatment, these DDR factors were initially localized inside nucleoli and then migrated to nucleolar caps (Figures 2 and 3C). RPA and TopBP1 enrichment indicated the presence of ssDNA stretches in nucleoli of the treated cells. Interestingly, proteins representing ATR and ATM signaling pathways almost completely co-localized inside nucleoli and later in nucleolar caps (Figure 3A). The amount of nucleoli-localized phospho-ATR decreased significantly by the third hour of hypoosmotic stress (Figure 3A) which was consistent with the decrease of the total amount of phospho-ATR under prolonged treatment (Figure 1I). To determine whether DDR signaling does originate from nucleoli and involves ribosomal genes, we performed ChIP with antibodies against γH2AX, TopBP1, RPA, Nbs1 and Mre11. All were enriched at rDNA sequences in HeLa cells under hypoosmotic conditions (Figure 3B). Notably, the highest enrichment was observed for RPA and TopBP1 at the 45S RNA gene promoter (Figure 3B). Co-localization analysis of γH2AX and UBF, Pol I transcription factor, showed that hypoosmotic stress-induced DDR signaling indeed originated from regions containing actively transcribed ribosomal genes (Figure 3C).

### Hypoosmotic stress induces R loop formation in nucleoli

**Figure 4.**
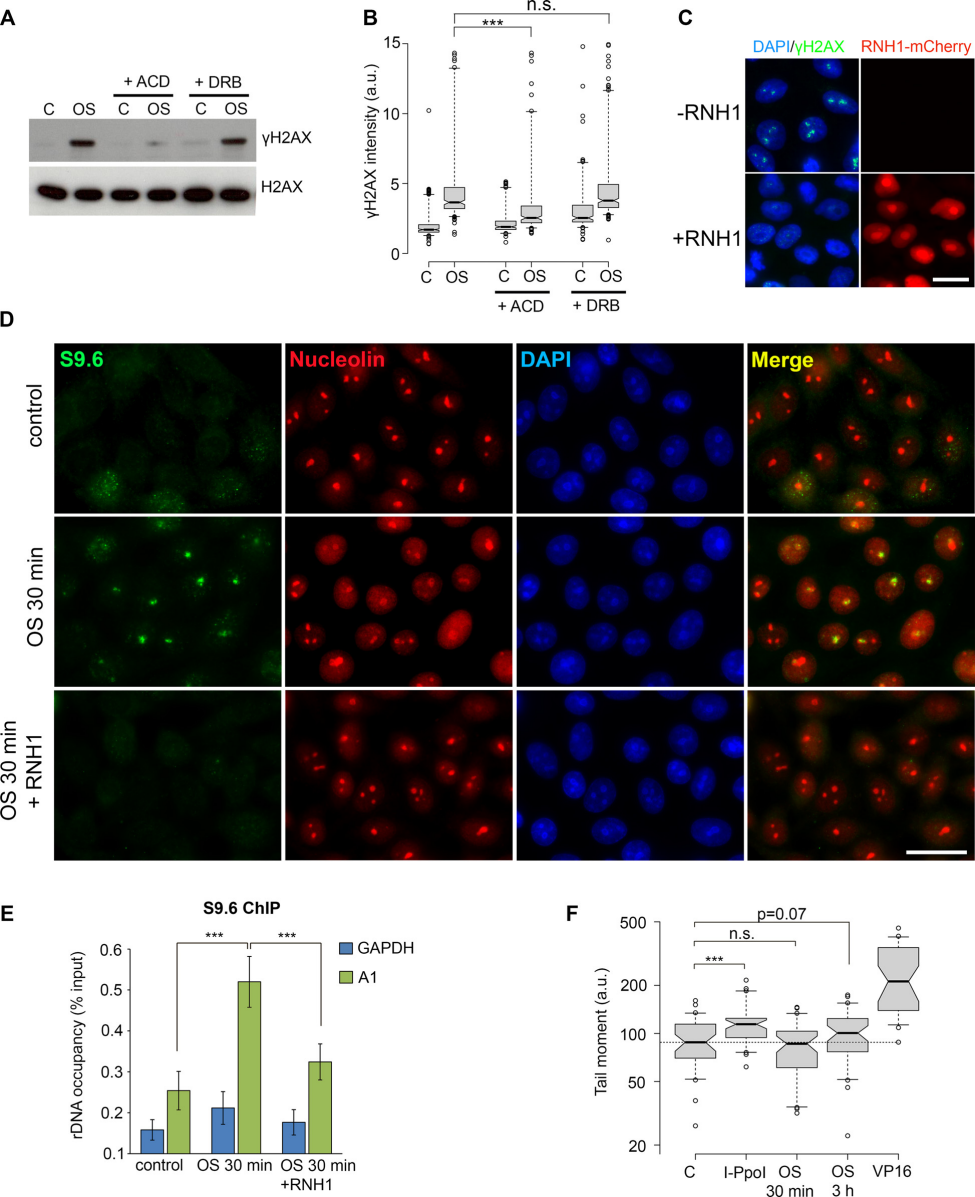
Hypoosmotic stress induces stabilization of R loops in nucleoli. (**A**) G1-phase HeLa cells were treated with either the Pol I inhibitor actinomycin D (ACD; 0.01 μg/ml) or RNA polymerase II inhibitor 5,6-dichlorobenzimidazole 1-β-D-ribofuranoside (DRB; 50 μM) for 3 h. During the last hour of the treatment the cells were subjected to hypoosmotic stress (OS). WB analysis of γH2AX was performed. Histone H2AX was used as a loading control. Control (C) represents HeLa cells that were not exposed to hypoosmotic stress. (**B**) Quantification of γH2AX fluorescence intensities in HeLa cells treated as in A. Box plots show the γH2AX intensities. Horizontal lines represent the median. ***P < 0.0001, n.s.—not significant (unpaired *t*-test, *n* > 500). (**C**) Control HeLa cells (−RNH1) and HeLa cells transiently overexpressing RNase H-mCherry fusion protein (+RNH1) were subjected to hypoosmotic stress for 1 h and stained for γH2AX (green). The red channel represents the fluorescence of RNase H-mCherry. Scale bar: 20 μm. (**D**) HeLa cells were subjected to hypoosmotic stress for 30 min (OS 30 min) and stained with antibodies against DNA:RNA hybrids (S9.6; green) and nucleolin (red). Lower panel shows immunostaining of the cells that were subjected to hypoosmotic stress and then treated with exogenous RNase H1 (OS 30 min + RNH1). Control represents HeLa cells that were not exposed to hypoosmotic stress (control). The DNA was stained with DAPI (blue). Scale bar: 20 μm. (**E**) R loop accumulation was measured by ChIP with the S9.6 antibody and qPCR for the rDNA region (A1: promoter) in control cells (control) or cells subjected to hypoosmotic stress for 30 min (OS 30 min). Part of the chromatin prepared from the cells subjected to hypoosmotic stress was treated with RNase H1 (OS 30 min + RNH1). The amplicon from the GAPDH gene was used as a negative control. Data are represented relative to the input. Values are the means ± SEM from at least three independent replicates (***P < 0.001, unpaired *t*-test). (**F**) G1-phase HeLa cells were subjected to hypoosmotic stress (OS) for 30 min or 3 h. HeLa cells expressing homing endonuclease I-PpoI that were treated with 4-hydroxytamoxifen for 4 h to activate I-PpoI (IPPO) and a topoisomerase II inhibitor etoposide-treated (10 μg/ml, 1 h) HeLa cells were used as positive controls (VP16). Control (C) represents untreated HeLa cells. The neutral comet assay was performed; box plots show the tail moment. Horizontal lines represent the median. ***P < 0.001, n.s.—not significant (unpaired *t*-test, *n* > 2000).

The unexpected activation of the ATR pathway in G1 cells lacking DNA replication along with the occupancy of ribosomal gene promoters with the ATR co-activator protein TopBP1 led us to hypothesize that R loops are sources of hypoosmotic stress-induced DDR (18,19). To check whether hypoosmotic stress-induced DDR depends on R loop stabilization, we, first, examined if inhibition of nucleolar transcription could impair DDR activation. For this purpose, we used two well-known small molecule inhibitors, actinomycin D (ACD), which, at low concentrations, inhibits Pol I-dependent nucleolar transcription, and 5,6-dichlorobenzimidazole 1-β-D-ribofuranoside (DRB), which primarily affects RNA polymerase II (Pol II) elongation (Supplementary Figure S5). Inhibition of nucleolar transcription by ACD completely abolished DDR signaling induced by hypoosmotic stress in G1 cells, whereas inhibition of Pol II elongation had no apparent effect (Figure 4A and B). Second, we overexpressed in cells the ribonuclease H (RNase H), which specifically degrades the RNA moiety in RNA:DNA hybrids to prevent R loop stabilization (20). Hypoosmotic stress-induced DDR was greatly diminished in HeLa cells overexpressing RNase H, indicating the crucial role of R loops in the DDR (Figure 4C). Direct evidence for the existence of nucleoli-associated R loops under hypoosmotic stress conditions has been provided by immunostaining analysis (Figure 4D) and ChIP (Figure 4E) with the RNA:DNA hybrid-specific antibody S9.6 (21). Both methods clearly demonstrated that hypoosmotic stress stabilized R loops in nucleoli.

It is generally thought that R loops can be converted into DSBs (22); however, it is questionable whether this happens under hypoosmotic stress conditions. A neutral comet assay did not show the appearance of DSBs in response to 30-min-long hypoosmotic stress (Figure 4F). Although long-term (3 h) hypoosmotic stress induced a shift in tail moment that was comparable to the one induced by the activity of homing endonuclease I-PpoI (most of its cleavage sites are located in rDNA), the statistical significance of these results was elusive (Figure 4F). These data suggest that hypoosmotic stress-induced activation of ATR- and ATM-mediated DDR does not depend on DSBs formation.

### Hypoosmotic stress-induced DDR leads to ATR/ATM-dependent silencing of rDNA transcription

**Figure 5.**
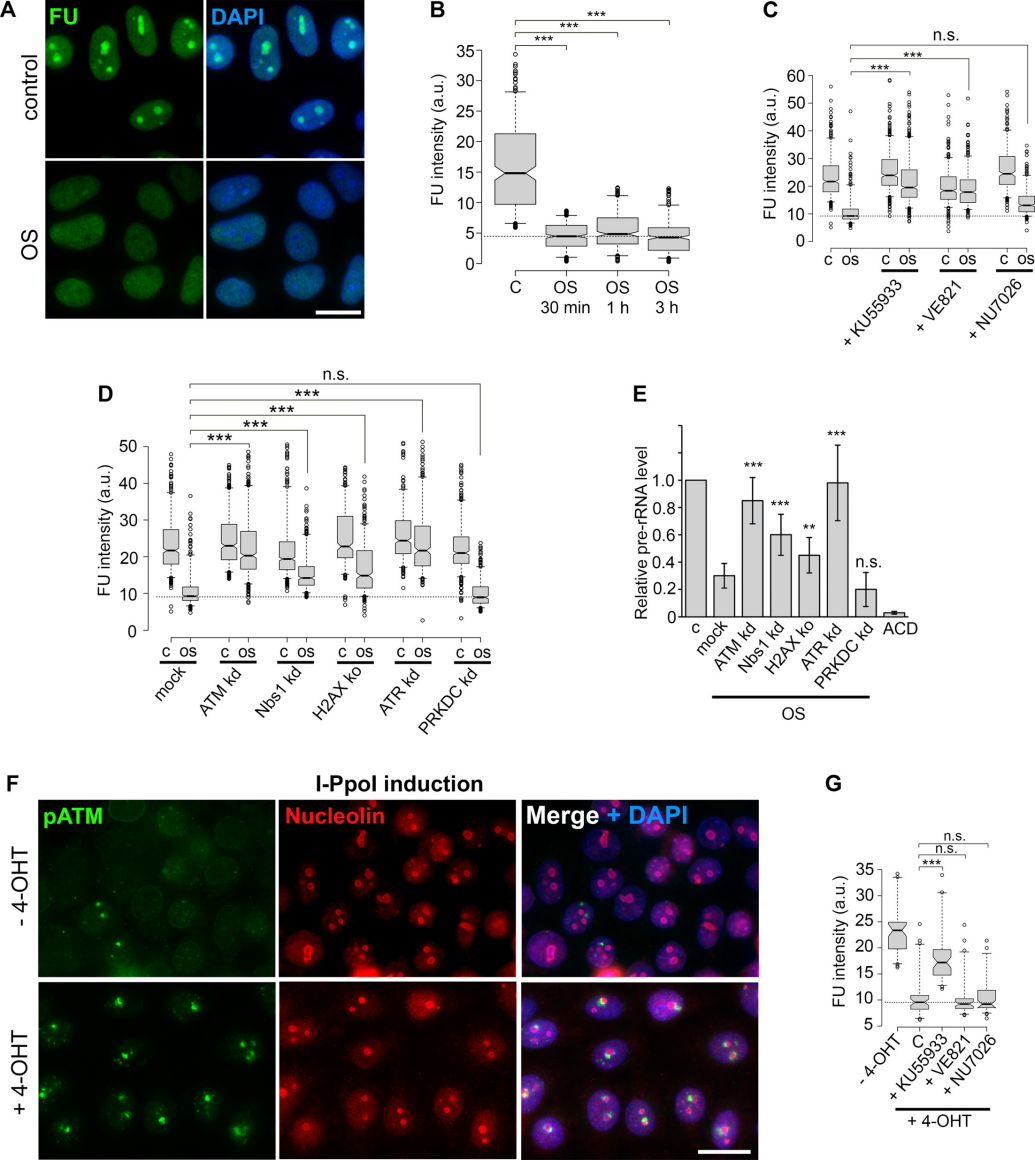
Hypoosmotic stress-induced DDR leads to ATR/ATM-dependent silencing of rDNA transcription. (**A**) HeLa cells, untreated or exposed to hypoosmotic stress for 3 h, were simultaneously pulsed with 5-fluorouridine (FU) for 3 h. Throughout the figure, FU was revealed by immunocytochemistry. The DNA was stained with DAPI (blue). Scale bar: 15 μm. (**B**) HeLa cells, untreated or exposed to hypoosmotic stress for 0.5, 1 or 3 h, were pulsed with FU for 30 min. Box plots show the FU fluorescence intensities. Horizontal lines represent the median. (**C**) Quantification of FU fluorescence intensities in HeLa cells that were pre-treated with specific inhibitors of either ATM (KU55933), ATR (VE821) or DNA-PKcs (NU7026) and then subjected to hypoosmotic stress for 3 h. Horizontal lines represent the median. ***P < 0.0001, *not significant (unpaired *t*-test, *n* > 500). (**D**) Quantification of FU fluorescence intensities in HeLa cells with CRISPR/Cas9-based knockout of histone H2AX or RNA interference-based knockdowns of either ATM, ATR, DNA-PKcs (PRKDC) or Nbs1 that were subjected to hypoosmotic stress for 3 h. Horizontal lines represent the median. ***P < 0.0001, n.s.—not significant (unpaired *t*-test, *n* > 500). (**E**) qRT-PCR showing levels of pre-rRNA normalized to GAPDH mRNA in HeLa cells treated as in D. HeLa cells treated with Pol I inhibitor ACD were used as a negative control. The data are represented as the mean ±SD. ***P < 0.0001, n.s.—not significant (unpaired *t*-test, *n* > 500). (**F**) HeLa cells expressing homing endonuclease I-PpoI were mock-treated (−4-OHT) or treated with 4-hydroxytamoxifen (+4-OHT) for 16 to activate I-PpoI and stained for γH2AX (green) and nucleolin (red). The DNA was stained with DAPI (blue). Scale bar: 20 μm. (**G**) HeLa cells expressing homing endonuclease I-PpoI were pre-treated with specific inhibitors of either ATM (KU55933), ATR (VE821) or DNA-PKcs (NU7026), and then treated with 4-hydroxytamoxifen (4-OHT) for 4 h to activate I-PpoI and pulsed with FU for 3 h. HeLa cells expressing I-PpoI, mock-treated (−4-OHT) or incubated with 4-OHT (C), were used as controls. Box plots show the FU fluorescence intensities. Horizontal lines represent the median. ***P < 0.0001, *not significant (unpaired *t*-test, *n* > 500).

Analysis of 5-FU incorporation into RNA showed that hypoosmotic stress conditions used in this study repressed nucleolar transcription (Figure 5A and B). To find out if nucleoli-specific DDR described here has a role in silencing of Pol I-dependent transcription, we analyzed HeLa cells treated with specific inhibitors of ATM, ATR and DNA-PKcs (Figure 5C). Inactivation of ATR and, to a lesser extent, ATM efficiently abrogated transcription silencing (Figure 5C). Further analysis performed using HeLa cells depleted for several DDR factors demonstrated that by their influence on stress-induced repression of nucleolar transcription they could be arranged as follows: ATR > ATM > Nbs1 > H2AX (Figure 5D and E).

To determine whether the involvement of both ATR and ATM in nucleolar transcription silencing is a unique property of hypoosmotic stress, we applied a well-known DSB-inducing system based on the utilization of homing endonuclease I-PpoI from *Physarum*. Most cleavage sites of this endonuclease are located in the 28S rRNA-coding region of rDNA, which makes it an ideal approach for the specific induction of nucleolar DNA damage (23). I-PpoI induction led to activation of the nucleoli-specific DDR, as shown by indirect immunofluorescence with an antibody against γH2AX (Figure 5F) and the silencing of Pol I-dependent transcription (Figure 5G). Nevertheless, for I-PpoI-induced DSBs, rDNA transcription inhibition was dependent only on ATM but not on ATR or DNA-PKcs (Figure 5G). Thus, while in case of rDNA-specific DSBs transcription silencing completely relies on ATM activity, in the hypoosmotic stress-treated cells ATR seems to be a key factor initiating transcriptional repression.

### ATR is an apical kinase in hypoosmotic stress-induced DDR

**Figure 6.**
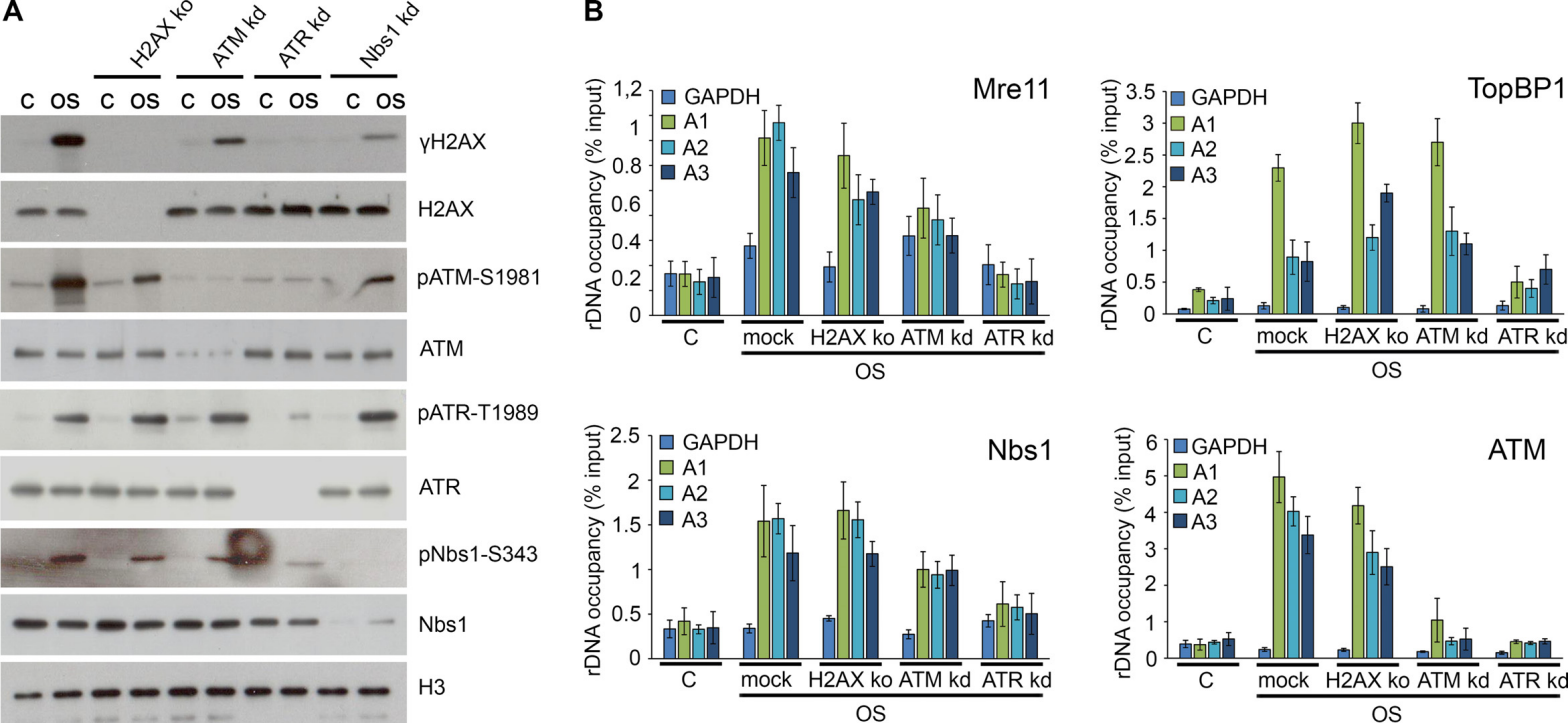
ATR is an apical kinase in hypoosmotic stress-induced DDR. (**A**) G1-phase HeLa cells with knockout of histone H2AX or knockdowns of either ATM, ATR or Nbs1 were subjected to hypoosmotic stress for 1 h. WB was performed with an antibodies against the indicated DNA repair factors. Histone H3 was used as a loading control. The control (C) represents untreated HeLa cells. (**B**) Occupancy of DNA repair factors at the rDNA of HeLa cells with knockout of histone H2AX or knockdowns of ATM or ATR under hypoosmotic stress conditions (1 h). ChIP with antibodies against ATM, Nbs1, Mre11 and TopBP1 followed by qPCR using the rDNA amplicons positioned as indicated on the scheme in Figure 3B (A1: promoter, A2: 5′-28S rRNA region, A3: 3′-28S rRNA region; the amplicon from GAPDH gene was used as a negative control). Data are represented relative to the input. Values are means ± SD from at least three independent replicates.

To further investigate the interdependence of different DDR factors in the course of cellular response to hypoosmotic stress, we analyzed their stress-induced activation in HeLa cells depleted for H2AX, ATM, ATR or Nbs1. This activation was monitored by the appearance of active phosphorylated forms of repair factors in cell extracts (phospho-ATM, phospho-ATR, phospho-Nbs1 and γH2AX) and/or by their localization in nucleoli (phospho-ATM, phospho-ATR, TopBP1 and phospho-Nbs1) in response to hypoosmotic stress. In ATR-depleted cells all signaling was absent, indicating that ATR is an apical kinase in hypoosmotic stress-induced DDR (Figure 6A and Supplementary Figure S6). DDR analysis in cells with downregulated Nbs1 led us to conclude that it acts downstream of ATR but is dispensable for ATM activation (Figure 6A). H2AX phosphorylation was significantly reduced in the ATM- and Nbs1-depleted cells, whereas in the ATR-depleted cells, it was completely abrogated (Figure 6A). In the H2AX knockout cells exposed to hypoosmotic stress ATR and Nbs1 activation were not compromised while ATM activation was partially suppressed (Figure 6A). To confirm and extend the analysis, we performed ChIP against ATM, Nbs1, Mre11 and TopBP1 in HeLa cells, depleted for either ATR, ATM or H2AX (Figure 6B). Only downregulation of ATR significantly affected the recruitment of its assistant factor TopBP1, the MRN complex subunits Nbs1 and Mre11, and ATM to rDNA in response to hypoosmotic stress (Figure 6B).

These results show that ATR activation serves as an event initiating DDR in response to hypoosmotic stress-induced R loops. The initial recruitment into nucleoli and activation of Nbs1 and ATM do not happen without ATR. Further reinforcement of ATM activation, which particularly is seen during 3 h-long treatment, is likely mediated by γH2AX-dependent positive feedback loop. The apical position of ATR in hypoosmotic stress-induced DDR clearly explains its influence on silencing of nucleolar transcription described here (see Figure 5D and E). Nevertheless, the facts that under hypoosmotic stress conditions ATM does not contribute to the recruitment/activation of other DDR factors (Figure 6A and B), but it is necessary for efficient silencing of nucleolar transcription (Figure 5D and E) suggest ATM to be an effector protein in stress-induced repression of nucleolar transcription. This may involve one of the known mechanisms of ATM-dependent Pol II transcription silencing (14−15).

### Activation of ATR and silencing of nucleolar transcription depend on TopBP1 and Treacle

**Figure 7.**
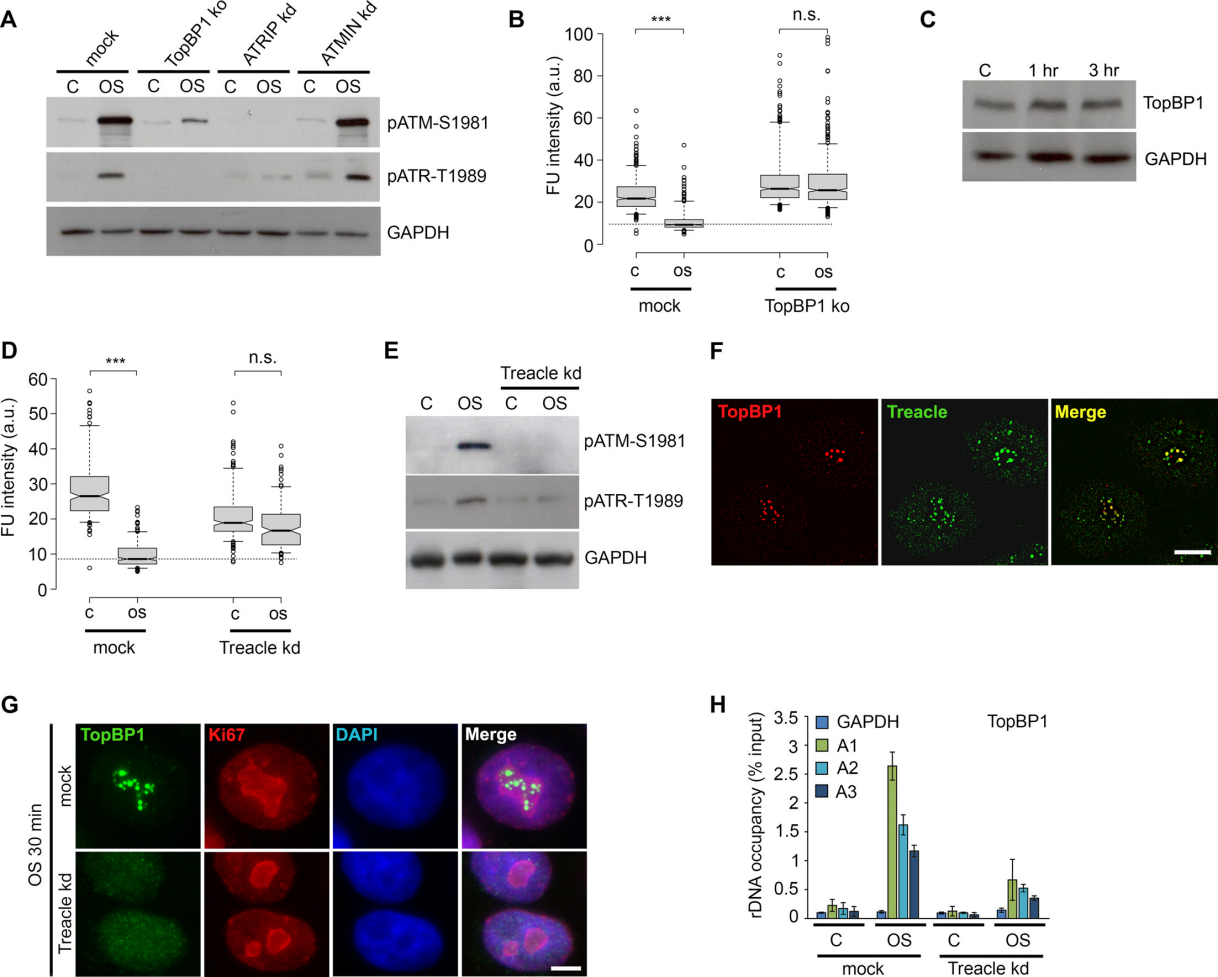
Activation of ATR and silencing of nucleolar transcription depend on TopBP1 and Treacle. (**A**) HeLa cells with knockout of TopBP1 or knockdowns of either ATRIP or ATMIN were subjected to hypoosmotic stress for 1 h (OS). WB was performed with antibodies against phospho-ATM (Ser1981) and phospho-ATR (Thr1989). GAPDH was used as a loading control. The control (C) represents untreated HeLa cells. (**B**) Intact HeLa cells and TopBP1 knockout HeLa cells were subjected to hypoosmotic stress (3 h; OS) and simultaneously pulsed with FU for 3 h. Throughout the figure, FU was revealed by immunocytochemistry. FU fluorescence intensities were quantified. The control (C) represents HeLa cells that were not subjected to hypoosmotic stress. Horizontal lines represent the median. ***P < 0.0001, n.s.—not significant (unpaired *t*-test, *n* > 500). (**C**) HeLa cells were subjected to hypoosmotic stress for 1 or 3 h. WB was performed with an antibody against TopBP1. GAPDH was used as a loading control. The control (C) represents untreated HeLa cells. (**D**) Intact HeLa cells and Treacle (TCOF1) knockdown HeLa cells were subjected to hypoosmotic stress (3 h; OS) and pulsed with FU for 3 h. FU fluorescence intensities were quantified. The control (C) represents HeLa cells that were not subjected to hypoosmotic stress. Horizontal lines represent the median. ***P < 0.0001, n.s.—not significant (unpaired *t*-test, *n* > 500). (**E**) Intact HeLa cells and Treacle (TCOF1) knockdown HeLa cells were subjected to hypoosmotic stress for 1 h (OS). WB was performed with antibodies against phospho-ATM (Ser1981) and phospho-ATR (Thr1989). GAPDH was used as a loading control. The control (C) represents untreated HeLa cells. (**F**) G1-phase HeLa cells were subjected to hypoosmotic stress (1 h), immunostained against TopBP1 (green) and Treacle (red) and analyzed with SIM microscopy. Scale bar: 5 μm. (**G**) Intact HeLa cells and Treacle (TCOF1) knockdown HeLa cells were subjected to hypoosmotic stress for 30 min and immunostained against TopBP1 (green) and nucleolar marker Ki67 (red). DNA was stained with DAPI (blue). Scale bar: 5 μm. (**H**) Occupancy of TopBP1 at the rDNA of Treacle knockdown HeLa cells under hypoosmotic stress conditions (1 h; OS). ChIP with an antibody TopBP1 followed by qPCR using the rDNA amplicons positioned as indicated on the scheme in Figure 3B (A1: promoter, A2: 5′-28S rRNA region, A3: 3′-28S rRNA region; the amplicon from GAPDH gene was used as a negative control). Data are represented relative to the input. Values are means ± SD from at least three independent replicates.

It is generally thought that TopBP1 is recruited to the ssDNA regions that are already occupied by ATR/ATRIP complex, and upon binding to the latter TopBP1 stimulates catalytic activity of ATR (24). Using HeLa cells depleted for TopBP1 and ATRIP we found that activation of ATR under hypoosmotic stress conditions strongly depended on these factors (Figure 7A). Expectedly, downregulation of TopBP1 expression strongly affected hypoosmotic stress-induced silencing of nucleolar transcription (Figure 7B). This clearly illustrates the necessity of induced DDR signaling for the process. Taking into account that overexpression of TopBP1 was shown to silence Pol I transcription in an ATR-dependent manner (25), we checked if hypoosmotic stress alters expression of TopBP1. No change in the expression level of TopBP1 was detected in hypoosmotic stress-treated cells (Figure 7C).

Another factor that was reported to participate in DNA damage-induced silencing of nucleolar transcription is Treacle (also known as TCOF1) (13,26), a nucleolar factor implicated in ribosome biogenesis and mutated in Treacher Collins syndrome (27). We performed analysis of transcription in Treacle-depleted cells and found that in the absence of Treacle the silencing of Pol I transcription under hypoosmotic stress conditions was compromised (Figure 7D). Unexpectedly, Treacle operated upstream of hypoosmotic stress-stimulated nucleolar DDR signaling as evidenced by a lack of ATR and ATM activation in Treacle knockdown cells (Figure 7E). Using indirect immunofluorescence analysis and ChIP we demonstrated that Treacle is indispensable for recruitment and/or retention of TopBP1 in nucleoli under hypoosmotic stress condition (Figure 7F–H). Noteworthy, Treacle was not necessary for TopBP1 recruitment and DDR activation in course of replication stress (Supplementary Figure S7). Although recruitment of TopBP1 to nucleoli in response to hypoosmotic stress seems to depend on the presence of ATR (see Figure 6A and Supplementary Figure S6), its retention and subsequent stimulation of ATR kinase activity need Treacle (see Figure 7E–H).

## DISCUSSION

Pol I-dependent transcription is downregulated in response to rDNA-specific DNA damage, as well as DNA breaks induced outside nucleoli (11−13). It is likely that the DNA damage-induced silencing of rDNA transcription relies on ATM activity (11−13). Alternatively, Grummt and colleagues presented evidence for DNA damage-independent silencing of nucleolar transcription that is provided by the NuRD chromatin remodeler, which is recruited to nucleoli by the rDNA-encoded lncRNA, *PAPAS*, upon long-term (4 h) hypoosmotic stress (28). The role of *PAPAS* in rDNA transcription silencing is verified (28,29); however, it is unclear whether it is sufficient for this process. The interconnection between the two mechanisms providing Pol I transcription inhibition has not been discussed elsewhere.

**Figure 8.**
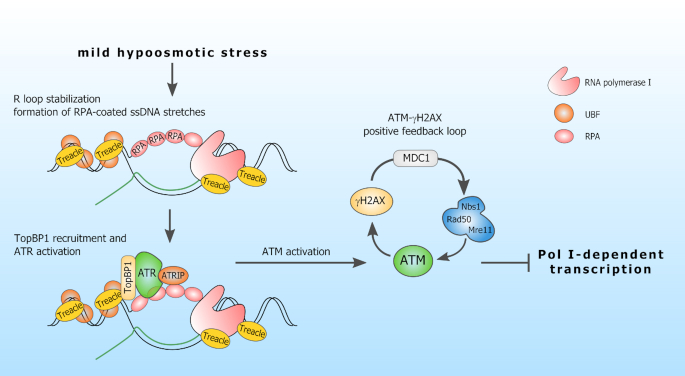
Model illustrating how hypoosmotic stress induces silencing of Pol I-dependent transcription.

Here, we provide evidence for a novel mechanism of stress-induced silencing of nucleolar transcription (Figure 8). We demonstrate that under hypoosmotic stress conditions, R loops in transcribed ribosomal genes are stabilized, thus, generating RPA-coated stretches of ssDNA. This results in the recruitment of ATR to nucleoli and its ATRIP- and TopBP1-dependent activation. Our data corroborate the idea that ATR can be activated by unpaired ssDNA sequences (18), which usually form at sites of R loops stabilization. Subsequent activation of ATM is mediated by ATR and does not depend on DSBs. ATR can act directly, as its ability to phosphorylate ATM was previously reported (6), or through the recruitment of the MRN complex. This recruitment may be based on the ability of Nbs1 to bind directly to the protein-coated ssDNA, the stabilization/generation of which is partially governed by ATR (30). γH2AX amplifies this DNA damage signal by generating a positive feedback loop involving an MDC1 protein and an MRN complex (31). As a result, ATM is activated extensively and shuts down nucleolar transcription probably through one of the known mechanisms of ATM-dependent Pol II transcription silencing (32,15).

The question is how the *PAPAS*-dependent pathway cooperates with the described one given that both are activated in response to hypoosmotic stress. First, in contrast to the study by Zhao *et al.* (28), we used G1-synchronized cells in part of the experiments. Most importantly, the treatment used in our study (50% DMEM/50% H2O for 0.5–3 h) is milder than one used by Zhao *et al.* (30% DMEM/70% H2O for 4 h) (28) and does not induce high expression of *PAPAS* (Supplementary Figure S8A). Consequently, depletion of the NuRD complex subunits (MTA2 and CHD4) does not substantially alter the repression of nucleolar transcription induced by mild hypoosmotic stress conditions used (Supplementary Figure S8B). At the same time, ATR/ATM-dependent pathway of Pol I transcription silencing operates in cells treated with hypoosmotic stress conditions used by Zhao *et al.* (28) as well. Moreover, transcription silencing in these cells is partly sensitive to ATR inhibition (data not shown). Apparently, this means that under hypoosmotic stress two different pathways of nucleolar transcription repression can act at once: the first is ATR/ATM-mediated and is induced by R loops stabilization; the second, PAPAS/NuRD-dependent one, becomes involved when the stress intensifies.

Unexpectedly, we found that Treacle, a nucleolar factor mutated in Treacher Collins syndrome, is needed for TopBP1 retention in nucleoli under hypoosmotic stress conditions. As a result, cells lacking Treacle cannot activate ATR and subsequent DDR signaling and cannot repress nucleolar transcription in response to mild hypoosmotic stress. Treacle is known to assist recruitment of Nbs1 to nucleoli during DNA damage (13,26), but its necessity for ToPB1 recruitment/retention in nucleoli is reported for the first time. Although it is tempting to suggest that TopBP1 interacts with Treacle by its BRCT domains as it happens in the case of Nbs1 (13,26), this should be further investigated.

Targeted DSB-induced rDNA transcription silencing (11,12) may simply reflect the necessity of transcription limitation to prevent the aggravation of DNA damage and to promote DNA repair. In this context, there are no conceptual differences between the inhibition of Pol I- or Pol II-dependent transcription (14−15). However, our data on hypoosmotic stress along with studies from other groups (13,28,29) suggest that the nucleolus and specifically the Pol I transcription machinery are an omni-purpose cell stress sensor. The higher intensity of Pol I-dependent compared to Pol II-dependent transcription makes it more vulnerable to stress factors, such as osmotic and heat stresses, reactive oxygen species, etc. Therefore, the stabilization of R loops and the activation of nucleoli-specific DDR in response to mild hypoosmotic stress appears natural. This property provides efficient rDNA transcription silencing and further molecular outcomes even prior to DSB generation. Nucleoli-specific DDR may initiate the cell stress response in multiple complementary ways. ATR and ATM can lead to activation of the checkpoint kinases and p53, thereby arresting cell cycle progression and/or inducing cell death. Moreover, p53 activation can be triggered by rDNA transcription silencing itself. Inhibition of Pol I-dependent transcription results in the release of ribosomal proteins that suppress the MDM2 protein, resulting in p53 stabilization/accumulation (33). Structural changes of nucleoli accompanying Pol I transcription inhibition can also result in redistribution to the nucleoplasm of nucleolar proteins, such as B23, nucleolin, or fibrillarin, which contribute to the cellular stress response (8,34,35). Our study brings additional evidence for the silencing of Pol I-dependent transcription as a major effector in the nucleolus-mediated cell stress response.

## Supplementary Material

gkz436_Supplemental_Files
