# Supplementary material for: Hypoosmotic stress induces R loop formation in nucleoli and ATR/ATM-dependent silencing of nucleolar transcription
Source: Nucleic Acids Res. 2019 May 22;47(13):6811–25. doi: 10.1093/nar/gkz436 (PMC6648358; doi:10.1093/nar/gkz436)
Supplement: gkz436_Supplemental_Files [file gkz436_supplemental_files.pdf]

Supplementary Information

Hypoosmotic stress induces R loop formation in nucleoli and ATR/ATM-dependent silencing of nucleolar transcription

Velichko et al.

- Supplementary Figures S1-8
- Supplementary Tables S1-5

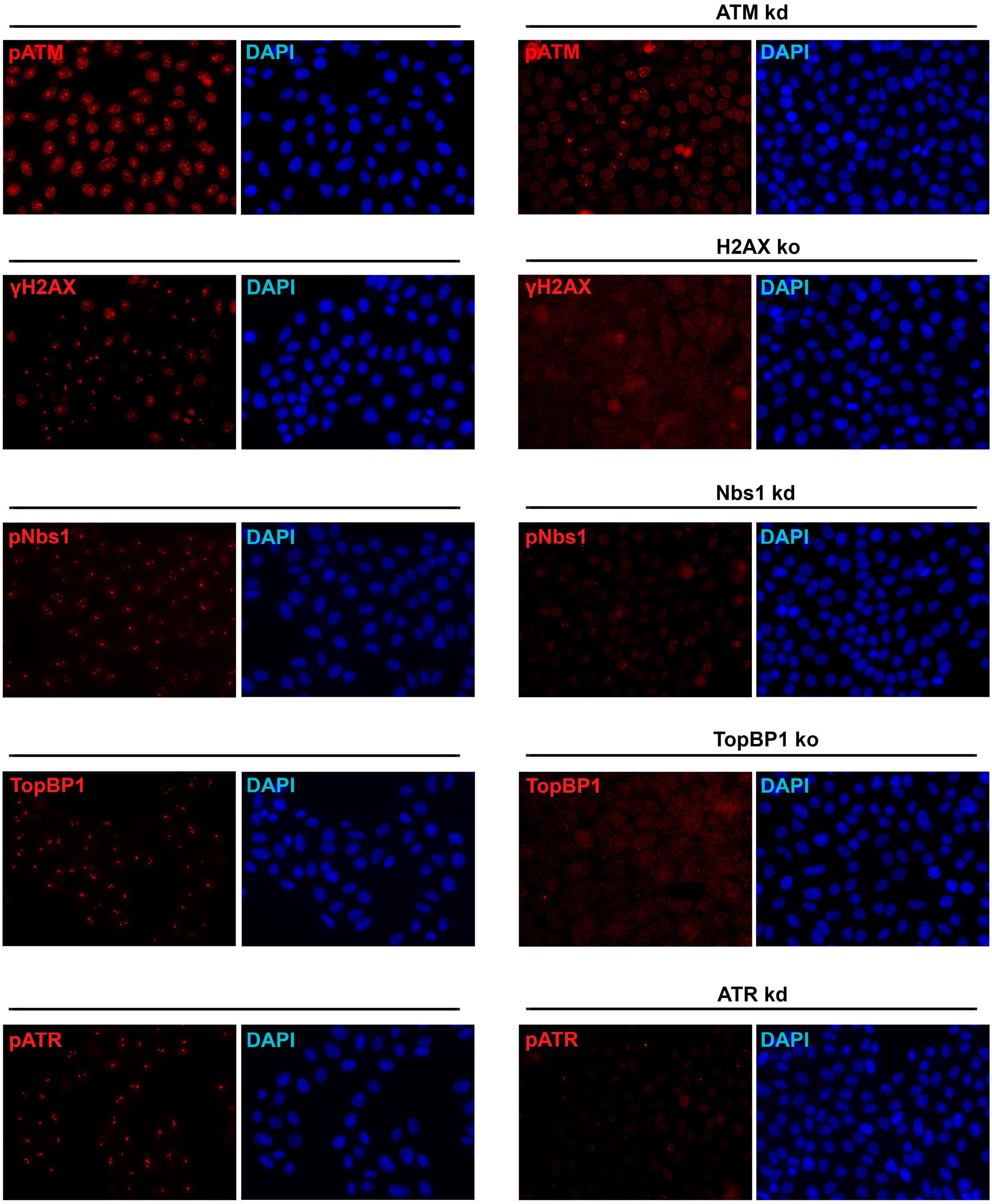

Supplementary Figure S1. Intact HeLa cells and HeLa cells with knockouts or knockdowns of the depicted on the figure DNA repair factors were subjected to hypoosmotic stress for 30 min and stained with corresponding phospho-specific antibody.

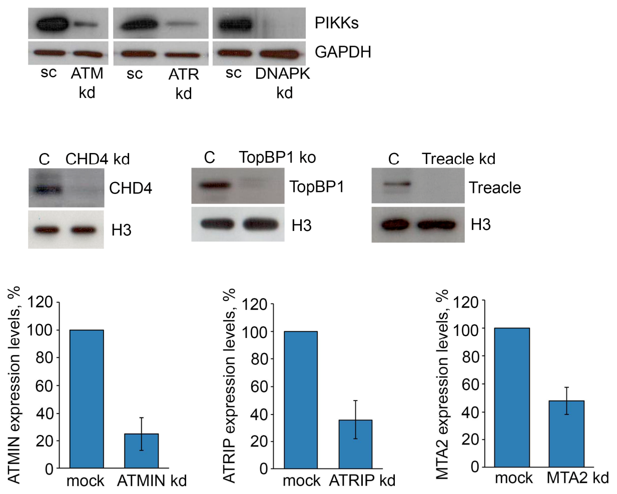

Supplementary Figure S2. HeLa cells were depleted for either ATM, ATR, DNA-PKcs, CHD4, MTA2, TopBP1, Treacle, ATMIN, or ATRIP using RNA interference or CRISPR/Cas9 technology. Knockdown and knockout efficiencies were analyzed by WB or qRT-PCR.

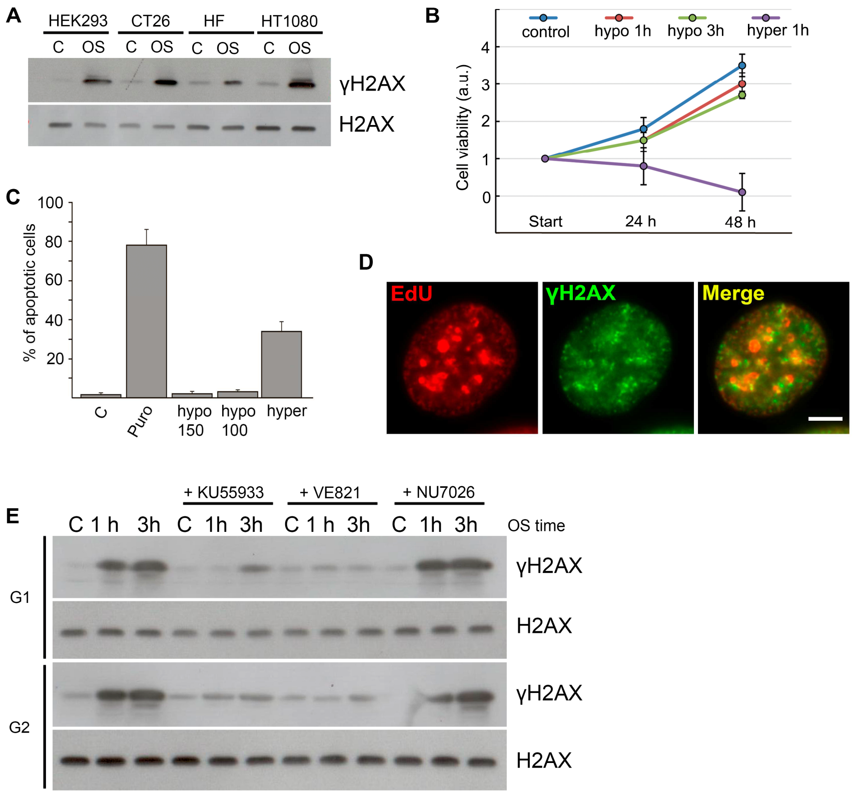

Supplementary Figure S3. Hypoosmotic stress induces phosphorylation of histone H2AX. (**A**) Human (HEK293T, HT1080, normal skin fibroblasts) and mouse (CT26) cells were treated with hypoosmotic stress (OS) for 3 h. WB analysis with antibodies against γH2AX was performed. Unmodified histone H2AX was used as a loading control. (**B**) Viability analysis of HeLa cells subjected to hypo- or hyperosmotic stress. Cells that were either untreated, treated with hypoosmotic stress (150 mOsm/L) for 1 or 3 hours (hypo), or treated with hyperosmotic stress (600 mOsm/L) for 1 hour (hyper) and allowed to recover for the indicated intervals (24 and 48 hours) were assayed for cellular viability using MTT reduction (the absorbance at 570 nm is directly proportional to cell number). Error bars represent the SEM. (**C**) HeLa cells, untreated or treated either with puromycin (puro; 10 µM, 16 h), hypoosmotic (hypo; 150 or 100 mOsm/L, 3 h), or hyperosmotic stress (hyper; 600 mOsm/L, 3 h), were analyzed using CellEvent Caspase 3/7 Detection Reagent. Percent of caspase 3/7-positive (apoptotic) cells is shown. (**D**) HeLa cells were pulse-labeled with EdU (10 µM, 30 min), subjected to hypoosmotic stress, and stained for γH2AX (green). EdU was revealed by Click Chemistry (red). Representative S-phase cell is shown. Scale bar: 5 µm. (**E**) WB analysis of γH2AX in G1- and G2-phase HeLa cells pretreated with specific inhibitors of either ATM (KU55933), ATR (VE821) or DNA-PKcs (NU7026) and subjected to hypoosmotic stress for 1 or 3 hours. Unmodified histone H2AX was used as a loading control.

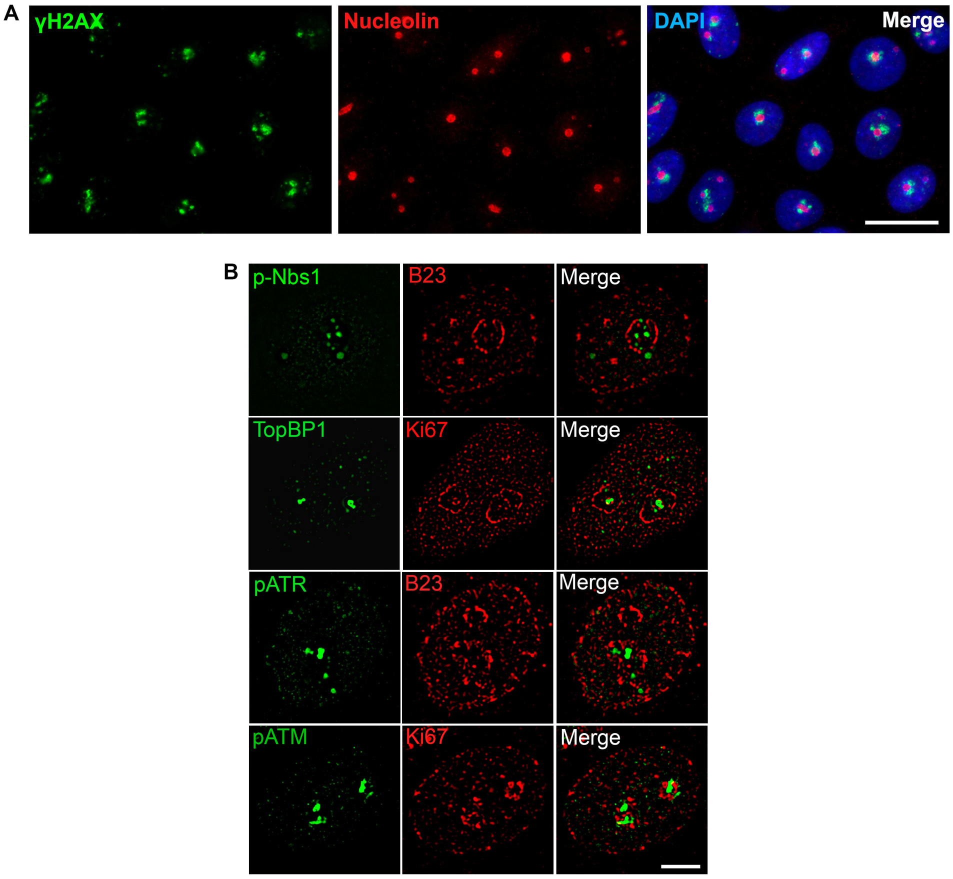

Supplementary Figure S4. Hypoosmotic stress leads to nucleoli-specific activation of ATR- and ATM-dependent signaling. (**A**) HeLa cells were subjected to hypoosmotic stress for 3 h and stained for γH2AX (green) and nucleolin (red). The DNA was stained with DAPI (blue). Scale bar: 20 µm. (**B**) Structured illumination microscopy (SIM) analysis of G1-phase HeLa cells that were treated with hypoosmotic stress for 1 h and stained for DDR factors (phospho-Nbs1 (Ser343), TopBP1, phospho-ATM (Ser1981) or phospho-ATR (Thr1989); green) and nucleolar proteins (B23 or Ki67; red). Scale bar: 5 µm.

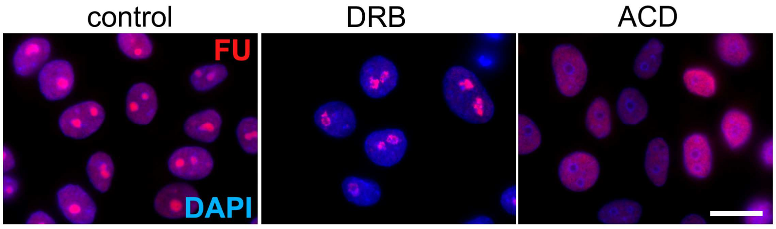

Supplementary Figure S5. HeLa cells, untreated or treated either with RNA polymerase II inhibitor 5,6-dichlorobenzimidazole 1-β-D-ribofuranoside (DRB; 50 μM, 3 h) or pol I inhibitor actinomycin D (ACD; 0.01 μg/ml, 3 h), were simultaneously pulsed with FU for 3 h. FU was revealed by immunocytochemistry (red); the DNA was stained with DAPI (blue). Scale bar: 20 μm.

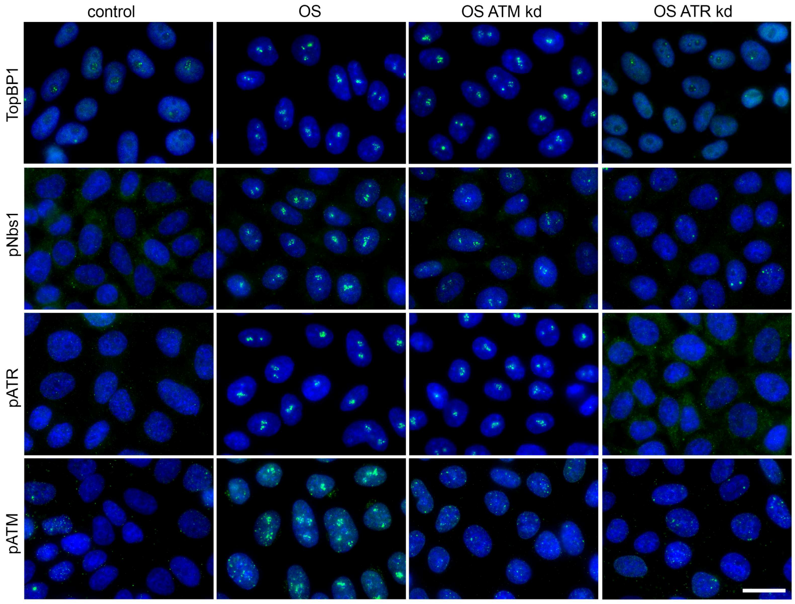

Supplementary Figure S6. HeLa cells depleted for ATM or ATR were subjected to hypoosmotic stress for 1 h and stained for either TopBP1, phospho-Nbs1 (Ser343), phospho-ATR (Thr1989) or phospho-ATM (Ser1981) (green). The DNA was stained with DAPI (blue); merged representative images are shown. Scale bar: 20 µm.

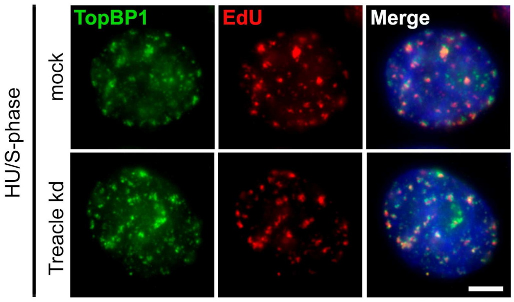

Supplementary Figure S7. Treacle is not necessary for TopBP1 recruitment during replication stress. Intact and Treacle knockdown HeLa cells were pulse-labeled with EdU (10 µM, 30 min), treated with hydroxyurea (20 mM, 1 h) and immunostained against TopBP1 (green). EdU was revealed by Click Chemistry (red); the DNA was stained with DAPI (blue). Representative S-phase cell is shown. Scale bar: 5 µm.

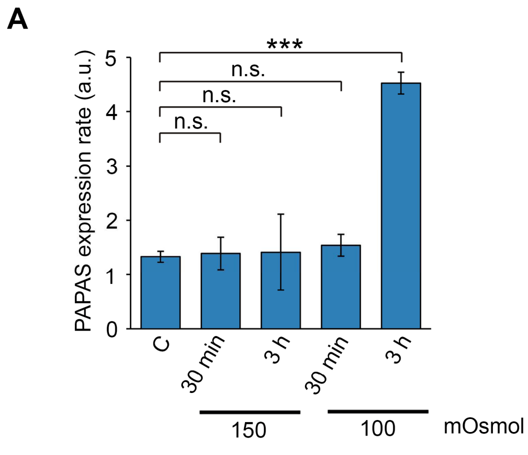

Supplementary Figure S8. Analysis of involvement of *PAPAS*/NuRD-dependent pathway in nucleolar transcription silencing induced by hypoosmotic stress conditions used in this study. (**A**) qRT-PCR showing levels of *PAPAS* lncRNA normalized to GAPDH mRNA in HeLa cells treated with different types of hypoosmotic stress (150 or 100 mOsm/L, 30 min or 3 h). The data are represented as the mean ±SD. ***P < 0.001, n.s. - not significant (**B**) Quantification of FU fluorescence intensities in HeLa cells depleted for CHD4 or MTA2 that were subjected to hypoosmotic stress for 3 h. Control (C) represents HeLa cells not treated with hypoosmotic stress. Horizontal lines represent the median.

Supplementary Tables

Supplementary Table S1		
	sense	antisense
MTA2 siRNA (custom-made set)	5’GGUGGGAGAUUACGUCUAUdTdT<br>5’CCGAGAUAUCACUCUGUUUdTdT<br>5’CCACCUACACUAAGCCAAAdTdT	5’AUAGACGUAAUCUCCCACCdTdT<br>5’AAACAGAGUGAUAUCUCGGdTdT<br>5’UUUGGCUUAGUGUAGGUGGdTdT
ATRIP siRNA (custom-made set)	5’GGAACAGAGAAGAUCACAUdTdT<br>5’GGGUCAUCCCUAAGCCUUUdTdT<br>5’GCCUAUCGCAGAAGGACAAdTdT	5’AUGUGAUCUUCUCUGUUCCdTdT<br>5’AAAGGCUUAGGGAUGACCCdTdT<br>5’UUGUCCUUCUGCGAUAGGCdTdT

Supplementary Table S2		
	For	Rev
gRNA1 H2AFX	5’ATGGCCAGCTGCAGGTGGCG	5’CGCCACCTGCAGCTGGCCAT
gRNA2 H2AFX	5’CGAGGAGCTCAACAAGCTGC	5’GCAGCTTGTTGAGCTCCTCG
gRNA1 TopBP1	5’CACCGAAACTGGATGTTCGGCTCT T	5’AAACAAGAGCCGAACATCCAGTTTC
gRNA2 TopBP1	5’CACCGATATATCTTTGCGGTTTTAG	5’AAACCTAAAACCGCAAAGATATATC

Supplementary Table S3		
	For	Rev
45S RNA for RT-qPCR	5’GCCTTCTCTAGCGATCTGAGAG	5’CCATAACGGAGGCAGAGACA
GAPDH for RT-qPCR	5’AAACTGTGGCGTGATGGC	5’CAGTGGGGACACGGAAGG
ATMIN for RT-qPCR	5’ACATGCACCTAGTCAAGAGCC	5’CATAAAGTGCTGTTTTACGAGAGA
ATRIP for RT-qPCR	5’CCCAGGTTCCAGTGTGTGTT	5’GAGCCACACCACCTCTTGTT
MTA2 for RT-qPCR	5’ATGAGACTATGGCAGGGGCA	5’AAACACCACAGGATTGGGGG

Supplementary Table S4	
Forward primer for RT-qPCR of PAPAS	5’TAATACGACTCACTATAGGGAGG
Reverse primer for RT-qPCR of PAPAS	5’GACGACAGGTCGCCAGAGGA
Primer for cDNA synthesis of PAPAS	5’TAATACGACTCACTATAGGGGAGGTATATCTTTCGCTCCGAG

Supplementary Table S5		
	For	Rev
A1 for ChIP	5' GGTATATCTTTCGCTCCGAGTC	5' ACAGGTCGCCAGAGGACAG
A2 for ChIP	5' GGTTGCTTGGGAATGCAG	5' CTTGTTGACTATCGGTCTCGTG
A3 for ChIP	5' TGGCGCTAAACCATTCGTAG	5'GTCGAGGGCTGACTTTCAATAG
GAPDH for ChIP	5’AAACTGTGGCGTGATGGC	5’CAGTGGGGACACGGAAGG
